# Metabolic tumor volume and total lesion glycolysis for predicting prognosis in diffuse large B-cell lymphoma: a retrospective PET/CT study

**DOI:** 10.3389/fonc.2026.1862170

**Published:** 2026-07-31

**Authors:** Yang You, Weifeng Zhang, Zhifei Zhen, Junling Xu, Ang Xuan

**Affiliations:** Department of Nuclear Medicine, Henan Provincial People’s Hospital/People’s Hospital of Zhengzhou University, Zhengzhou, China

**Keywords:** ^18^F-FDG PET/CT, diffuse large B-cell lymphoma, international prognostic index, metabolic tumor volume, prognosis, survival, total lesion glycolysis

## Abstract

**Background:**

Diffuse large B-cell lymphoma (DLBCL) is characterized by marked clinical heterogeneity, and conventional prognostic indices provide limited individualized risk prediction. We investigated the prognostic value of baseline ^18^F-FDG PET/CT-derived volumetric metabolic parameters in newly diagnosed DLBCL.

**Methods:**

This retrospective study included 176 consecutive patients with histologically confirmed DLBCL who underwent baseline ^18^F-FDG PET/CT before first-line R-CHOP or R-CHOP-like immunochemotherapy between January 2022 and December 2024. Metabolic tumor volume (MTV) was measured using a 41% SUVmax threshold, and total lesion glycolysis (TLG) was calculated as MTV × SUVmean. Optimal cutoff values were determined by ROC analysis. Survival outcomes were evaluated using Kaplan-Meier analysis and Cox proportional hazards models. Predictive performance was assessed by Harrell’s C-index.

**Results:**

The median age was 58 years, and 54.5% of patients were male. After a median follow-up of 26.4 months, 48 patients experienced progression; including 5 deaths without documented progression, the composite progression-free survival (PFS) endpoint comprised 53 events, and 30 patients died. The optimal cutoffs were 274.8 cm³ for MTV and 1,886.3 g for TLG. High MTV was associated with significantly inferior 2-year PFS (50.7% vs 79.9%; P<0.001) and OS (71.3% vs 92.8%; P<0.001). In multivariable analysis, high MTV remained the only significant independent predictor of PFS (HR 3.16, 95% CI 1.82-5.50; P<0.001). Incorporating MTV into the IPI improved discrimination compared with IPI alone (C-index 0.697 vs 0.567).

**Conclusions:**

In this single-center cohort, baseline MTV was a robust independent prognostic biomarker for PFS that substantially enhanced risk stratification beyond the IPI; its association with OS was exploratory given the limited number of events. Integration of volumetric PET/CT metrics into prognostic models may facilitate more individualized therapeutic decision-making. The optimism-prone, internally derived cutoff requires external validation; prospective, multicenter validation with standardized measurement protocols is warranted.

## Introduction

Diffuse large B-cell lymphoma (DLBCL) accounts for approximately 30% to 40% of newly diagnosed non-Hodgkin lymphomas and represents the single most common aggressive lymphoma subtype worldwide (1, 2). Although the addition of rituximab to cyclophosphamide, doxorubicin, vincristine, and prednisone (R-CHOP) has markedly improved outcomes, roughly one-third of patients still experience primary refractory disease or relapse within the first two years, portending a substantially worse prognosis (3, 4). This clinical heterogeneity underscores the pressing need for reliable biomarkers capable of identifying high-risk patients at diagnosis, thereby enabling risk-adapted therapeutic strategies.

The International Prognostic Index (IPI), originally developed in 1993 and subsequently refined as the National Comprehensive Cancer Network IPI (NCCN-IPI) (5, 6), remains the standard tool for prognostic stratification in DLBCL. However, these indices rely exclusively on clinical and laboratory parameters and offer only moderate discriminatory power in the rituximab era, with reported concordance indices of 0.55 to 0.65 (7). Cell-of-origin classification by immunohistochemistry, particularly the distinction between germinal center B-cell (GCB) and non-GCB (activated B-cell) subtypes via the Hans algorithm (8), and double-expressor lymphoma (DEL) status (9), provide complementary biological insights but still leave substantial prognostic uncertainty.

^18^F-Fluorodeoxyglucose positron emission tomography/computed tomography (^18^F-FDG PET/CT) has become indispensable in the management of DLBCL, serving integral roles in initial staging, interim response assessment, and end-of-treatment evaluation per the Lugano classification (10). Beyond its established qualitative utility, growing attention has centered on quantitative PET-derived volumetric parameters, including metabolic tumor volume (MTV) and total lesion glycolysis (TLG). MTV, defined as the aggregate volume of metabolically active tumor, captures the three-dimensional extent of disease burden, whereas TLG, the product of MTV and mean standardized uptake value (SUVmean), simultaneously encodes both volumetric and metabolic intensity information (11).

Multiple large-scale retrospective analyses have established that elevated baseline MTV and TLG are independently associated with inferior progression-free survival (PFS) and overall survival (OS), with hazard ratios frequently exceeding 3.0 for both endpoints (12). The LOTIS-2 clinical trial and RELEVANCE study corroborated these findings in prospective cohorts (13, 14), and the recently proposed International Metabolic Prognostic Index (IMPI) integrates MTV with clinical variables to yield prognostic discrimination surpassing that of the conventional IPI (15, 16). Despite this accumulating evidence, clinical adoption has been hampered by methodological heterogeneity in MTV measurement, including variations in segmentation thresholds (41% SUVmax, SUV ≥ 2.5, or gradient-based methods), absence of standardized cutoff values, and insufficient external validation (17, 18). An international benchmark initiative published in the Journal of Nuclear Medicine in 2024 has begun to address these standardization concerns (19).

Against this backdrop, the present study aimed to evaluate the prognostic value of baseline MTV and TLG derived from ^18^F-FDG PET/CT for predicting PFS and OS in a contemporary cohort of patients with newly diagnosed DLBCL treated with R-CHOP-based immunochemotherapy. We further sought to determine whether the incorporation of MTV into the IPI framework enhances prognostic accuracy and to explore the consistency of MTV-based risk stratification across clinically relevant subgroups, including cell-of-origin subtypes and IPI risk categories.

## Methods

### Study design and patient selection

**Figure 1:**
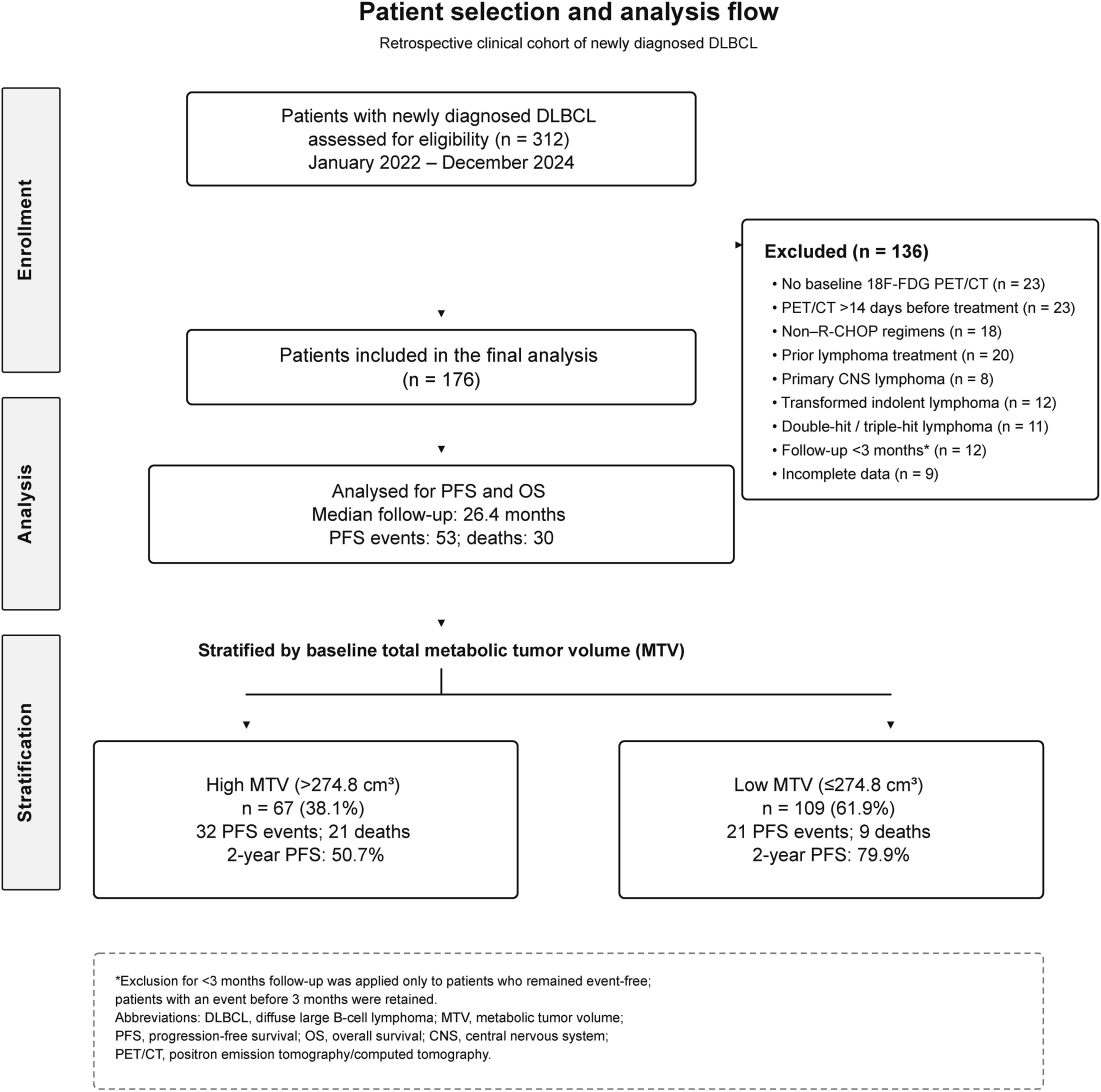
Patient selection flow diagram. A total of 312 patients diagnosed with diffuse large B-cell lymphoma (DLBCL) between January 2022 and December 2024 were screened. After sequential exclusion of patients without baseline ^18^F-FDG PET/CT (n = 23), PET/CT performed more than 14 days before treatment initiation (n = 23), non-R-CHOP regimens (n = 18), prior lymphoma treatment (n = 20), primary central nervous system lymphoma (n = 8), transformed indolent lymphoma (n = 12), double-hit/triple-hit lymphoma (n = 11), follow-up less than 3 months (n = 12), and incomplete data (n = 9), 176 patients were included and stratified into high-MTV (n = 67, 38.1%) and low-MTV (n = 109, 61.9%) groups using the optimal cutoff of 274.8 cm³.

This single-center retrospective observational study enrolled consecutive patients with newly diagnosed DLBCL at our institution between January 2022 and December 2024. The study protocol was approved by the institutional review board, and the requirement for written informed consent was waived given the retrospective nature of the investigation. Potential sources of bias were systematically considered: selection bias was mitigated by enrolling consecutive patients meeting predefined criteria; information bias was minimized through standardized PET/CT acquisition protocols and blinded image analysis by two independent readers; and confounding was addressed through multivariate Cox regression adjusting for clinically relevant covariates. The study was conducted in accordance with the Declaration of Helsinki and reported in compliance with the Strengthening the Reporting of Observational Studies in Epidemiology (STROBE) guidelines and its extensions (20). The patient selection process is detailed in **Figure 1**.

Inclusion criteria were as follows: (1) histopathologically confirmed DLBCL according to the World Health Organization (WHO) 2016 classification, which was the framework in routine diagnostic use at our institution throughout the enrolment period (the implications of the WHO 5th edition [2022] and the International Consensus Classification [2022] are considered in the Discussion); (2) availability of baseline ^18^F-FDG PET/CT performed within 14 days before initiation of first-line therapy; (3) treatment with R-CHOP or R-CHOP-like immunochemotherapy; and (4) adequate clinical and follow-up data. Patients with incomplete baseline PET/CT data or fewer than 3 months of follow-up were excluded; this minimum follow-up requirement was applied only to patients who remained event-free, whereas patients who experienced progression, relapse, or death within the first 3 months were retained (accounting for the minimum observed follow-up of 2.5 months). All included patients had complete data for the primary analysis variables; no imputation was required. Patients with primary central nervous system lymphoma, transformed indolent lymphoma, or high-grade B-cell lymphoma with MYC and BCL2 and/or BCL6 rearrangements (double-hit/triple-hit lymphoma) were excluded.

### Clinical data collection

Baseline demographic, clinical, laboratory, and pathological data were extracted from the electronic medical record. Variables included age, sex, Ann Arbor stage, Eastern Cooperative Oncology Group performance status (ECOG PS), presence of B symptoms, number of extranodal sites, serum lactate dehydrogenase (LDH), beta-2 microglobulin (β2-MG), and albumin. The IPI and NCCN-IPI scores were calculated according to established algorithms (5, 6). Cell-of-origin was classified as GCB or non-GCB by the Hans immunohistochemistry algorithm (CD10, BCL6, MUM1).8 Double-expressor status was defined as concurrent c-MYC (≥40% positive cells) and BCL2 (>50% positive cells) protein expression (9). The Ki-67 proliferation index was recorded for all patients.

### ^18^F-FDG PET/CT acquisition and analysis

Baseline ^18^F-FDG PET/CT scans were acquired on two PET/CT systems: a uEXPLORER total-body PET/CT scanner (United Imaging Healthcare Co., Ltd., Shanghai, China; n = 92) and a Discovery VCT PET/CT scanner (GE Healthcare, USA; n = 84), following a standardized institutional protocol. The scanner-specific case counts reflect institutional imaging records, and the reconstruction parameters for both systems are summarized in **Supplementary Table S1**. Both systems underwent routine quality control, and a uniform 41% SUVmax segmentation threshold was applied across scanners to mitigate inter-system variability in volumetric quantification. Reconstruction followed vendor-specific iterative algorithms consistent with EANM procedure guidelines; formal cross-vendor EARL harmonization was not performed, a limitation addressed in the Discussion. Patients fasted for at least 6 hours, and blood glucose was confirmed to be below 11.1 mmol/L (200 mg/dL) before injection. Weight-based ^18^F-FDG was administered intravenously at approximately 3.7 MBq/kg (median 4.57 MBq/kg; range, 2.62-7.81 MBq/kg; absolute range, 185–400 MBq), with image acquisition commencing approximately 60 minutes post-injection.

All PET/CT images were analyzed by two experienced nuclear medicine physicians blinded to clinical outcomes. Segmentation was performed independently, and discrepancies were resolved by consensus to yield a single agreed measurement per patient. Because only the consensus dataset was retained, a quantitative inter-reader agreement metric (e.g., intraclass correlation coefficient) could not be derived *post hoc*; the potential influence of inter-observer variability, which persists even with standardized 41% SUVmax thresholds, is acknowledged as a limitation. MTV was defined as the total volume of tumor tissue with standardized uptake value (SUV) exceeding 41% of the maximum SUV (SUVmax) for each lesion, in accordance with European Association of Nuclear Medicine (EANM) recommendations.17 All discrete hypermetabolic foci consistent with lymphoma involvement were individually segmented, and the total MTV (TMTV) was calculated as the sum of individual lesion volumes. TLG was computed as the product of MTV and SUVmean across all segmented lesions. SUVmax, SUVmean, and SUVpeak were recorded for each patient. Physiological uptake in the brain, heart, kidneys, bladder, and ureters was excluded from volumetric measurements. When physiological tracer activity in organs such as the adrenal glands, spleen, or liver was adjacent to or overlapped suspected lymphomatous involvement, lesions were disambiguated by joint review of the co-registered CT component, comparison with expected physiological biodistribution, and reader consensus; equivocal foci were classified as non-lymphomatous unless supported by a corresponding structural abnormality.

### Treatment and response assessment

All patients received first-line immunochemotherapy with R-CHOP (n = 136, 77.3%), dose-adjusted etoposide, prednisone, vincristine, cyclophosphamide, doxorubicin, and rituximab (R-EPOCH; n = 28, 15.9%), or other R-CHOP-like regimens (n = 12, 6.8%). The number of treatment cycles ranged from 4 to 8, with consolidation radiotherapy administered to 45 patients (25.6%) per institutional protocols. Treatment response was assessed according to Lugano 2014 criteria (10), with end-of-treatment evaluation comprising both PET/CT (Deauville 5-point scale) and conventional imaging.

### Outcomes and follow-up

The primary endpoints were PFS, defined as the time from diagnosis to disease progression, relapse, death from any cause, or last follow-up, and OS, defined as the time from diagnosis to death from any cause or last follow-up. Patients who were alive without progression at the time of last follow-up were censored.

### Statistical analysis

Continuous variables were expressed as median and interquartile range (IQR) and compared using the Mann-Whitney U test. Categorical variables were reported as frequencies and percentages and compared using the chi-square test or Fisher exact test, as appropriate. Receiver operating characteristic (ROC) curves were constructed to evaluate the discriminatory capacity of MTV, TLG, and SUVmax for predicting 2-year PFS events, and the area under the curve (AUC) was reported. Optimal cutoff values were determined by maximizing the Youden index (sensitivity + specificity - 1).

Survival curves were generated using the Kaplan-Meier method and compared with the log-rank test. Univariate Cox proportional hazards regression was performed for all candidate prognostic variables, and those achieving P < 0.10 were entered into a multivariate model using backward stepwise selection with a removal threshold of P > 0.10. A liberal screening threshold of P < 0.10 (rather than P < 0.05) was adopted deliberately to avoid prematurely excluding potential confounders, in accordance with established prognostic-modelling guidance. The proportional hazards assumption was verified for all covariates using scaled Schoenfeld residuals and the global goodness-of-fit test; no significant violations were detected (all P > 0.05). Given the near-collinearity between MTV and TLG (Spearman r = 0.923), only one volumetric parameter could enter the multivariate model; MTV was retained in preference to TLG because it showed marginally superior univariable discrimination (AUC 0.685 vs 0.663) and is the more widely reported and computationally simpler parameter, facilitating external comparability. Discriminatory performance of prognostic models was quantified with Harrell concordance index (C-index). Subgroup analyses evaluated the consistency of MTV prognostic value across predefined clinical strata. All statistical analyses were performed in R version 4.5.0 (R Foundation for Statistical Computing; packages survival, rms, pROC, survIDINRI, nricens, and Hmisc) and in Python 3.11 (lifelines v0.27, scikit-learn v1.3, and SciPy v1.11). Based on the rule of approximately 10 events per predictor variable (EPV) for Cox regression, the 53 PFS events observed in this cohort (48 progressions plus 5 deaths without prior progression) supported the inclusion of up to approximately 5 covariates in the multivariate PFS model. The 30 OS events limited the multivariate OS analysis to exploratory status with a single predictor. To guard against optimism arising from data-driven cutoff selection and model fitting within a single cohort, the C-index of each model was internally validated by bootstrap resampling (1,000 replications), and optimism-corrected C-indices and an optimism-corrected hazard ratio for high MTV were derived. In this primary optimism correction the MTV cutoff was held fixed at the value derived in the full cohort; as an additional sensitivity analysis the Youden-optimal cutoff was re-derived within each bootstrap replication to capture the optimism arising from data-driven threshold selection. The robustness of the MTV-PFS association to imaging platform was further examined by adding scanner model as a covariate, by stratifying the Cox model on scanner, and by testing an MTV-by-scanner interaction. As sensitivity analyses, MTV was additionally modelled as a continuous variable (log-transformed and with restricted cubic splines [3 knots]); the multivariate PFS model was repeated with first-line regimen (R-CHOP vs intensified) and consolidation radiotherapy as additional covariates and within the R-CHOP-only subgroup (n = 136). Differences between nested C-indices were tested by bootstrap with 95% confidence intervals; the incremental discrimination of adding MTV to the IPI was further quantified by the continuous net reclassification improvement (NRI) and the integrated discrimination improvement (IDI), and pairwise differences between ROC curves were compared using the DeLong test. Two-sided P values < 0.05 were considered statistically significant.

## Results

### Patient characteristics

A total of 176 patients with newly diagnosed DLBCL were included in the analysis (**Figure 1** presents the patient selection flow diagram). Baseline demographic and clinical characteristics are summarized in **Table 1**. The median age at diagnosis was 58 years (IQR, 49-66; range, 27-82), and 96 patients (54.5%) were male. Advanced-stage disease (Ann Arbor stage III-IV) was present in 113 patients (64.2%), and 44 patients (25.0%) had an ECOG PS of 2 or higher. The IPI score was 3 or higher (high-intermediate to high risk) in 66 patients (37.5%). Non-GCB subtype by the Hans algorithm was identified in 96 patients (54.5%), and 29 patients (16.5%) exhibited the double-expressor phenotype. The median serum LDH was 264 U/L (IQR, 211-339), and LDH was elevated above the upper limit of normal in 95 patients (54.0%).

**Table 1:** Baseline patient characteristics by MTV group.

Variable	Total (N = 176)	High MTV (n = 67)	Low MTV (n = 109)	P value
Age, median (IQR), y	58 (49-66)	58 (49-65)	57 (49-66)	0.822
Male sex, n (%)	96 (54.5)	36 (53.7)	60 (55.0)	0.989
Ann Arbor stage III-IV, n (%)	113 (64.2)	41 (61.2)	72 (66.1)	0.623
B symptoms, n (%)	68 (38.6)	25 (37.3)	43 (39.4)	0.902
ECOG PS ≥ 2, n (%)	44 (25.0)	22 (32.8)	22 (20.2)	0.089
Extranodal sites ≥ 2, n (%)	49 (27.8)	22 (32.8)	27 (24.8)	0.320
Bulky disease, n (%)	36 (20.5)	16 (23.9)	20 (18.3)	0.489
IPI score ≥ 3, n (%)	66 (37.5)	29 (43.3)	37 (33.9)	0.279
LDH elevated, n (%)	95 (54.0)	42 (62.7)	53 (48.6)	0.097
LDH, median (IQR), U/L	264 (211-339)	288 (222-385)	248 (200-310)	0.032
β2-MG, median (IQR), mg/L	2.46 (1.85-3.20)	2.48 (1.98-3.38)	2.38 (1.65-3.07)	0.246
Albumin, median (IQR), g/L	38.0 (34.4-41.5)	37.2 (32.8-41.0)	38.6 (35.2-42.0)	0.118
Hemoglobin, median (IQR), g/L	122.2 (107.4-136.2)	118.5 (104.0-133.0)	124.8 (110.2-138.0)	0.065
Pathological features				
Non-GCB subtype, n (%)	96 (54.5)	43 (64.2)	53 (48.6)	0.063
DEL status, n (%)	29 (16.5)	13 (19.4)	16 (14.7)	0.541
Ki-67, median (IQR), %	71 (59-83)	71 (59-85)	70 (59-80)	0.492
BCL2 positive, n (%)	103 (58.5)	42 (62.7)	61 (56.0)	0.454
c-MYC positive, n (%)	51 (29.0)	22 (32.8)	29 (26.6)	0.464
PET/CT parameters				
SUVmax, median (IQR)	16.55 (12.90-22.62)	17.90 (13.30-23.35)	16.10 (12.70-20.80)	0.149
SUVmean, median (IQR)	8.15 (5.80-10.90)	8.60 (6.30-11.50)	7.80 (5.50-10.40)	0.138
MTV, median (IQR), cm³	186.40 (95.17-408.60)	483.10 (346.50-762.40)	111.60 (66.40-186.30)	<0.001
TLG, median (IQR), g	1524.80 (710.52-3818.93)	4940.30 (2951.15-9846.25)	908.80 (492.00-1378.80)	<0.001
Treatment regimen				
R-CHOP, n (%)	136 (77.3)	48 (71.6)	88 (80.7)	0.384
R-EPOCH, n (%)	28 (15.9)	13 (19.4)	15 (13.8)	
R-CHOP-like, n (%)	12 (6.8)	6 (9.0)	6 (5.5)	
Consolidation RT, n (%)	45 (25.6)	19 (28.4)	26 (23.9)	0.594

MTV, metabolic tumor volume; IQR, interquartile range; ECOG PS, Eastern Cooperative Oncology Group performance status; IPI, International Prognostic Index; LDH, lactate dehydrogenase; β2-MG, beta-2 microglobulin; GCB, germinal center B-cell; DEL, double-expressor lymphoma; SUVmax, maximum standardized uptake value; TLG, total lesion glycolysis. Because the MTV grouping is derived from the survival outcome via the Youden-optimal cutoff, between-group P values should be interpreted descriptively rather than inferentially; standardized mean differences for selected variables were MTV 1.46, TLG 1.22, LDH ratio 0.36, non-GCB 0.32, and ECOG PS 0.30.

The overall response rate to first-line therapy was 87.5% (154/176), with a complete response (CR) rate of 53.4% (94/176). CR was defined as an end-of-treatment Deauville score of 1–3 per the Lugano 2014 criteria; the end-of-treatment Deauville distribution was score 1, n = 12; score 2, n = 36; score 3, n = 46; score 4, n = 60; and score 5, n = 22. After a median follow-up of 26.4 months (range, 2.5-36.0), 48 patients (27.3%) experienced disease progression or relapse; including 5 additional deaths without documented progression, the composite PFS endpoint comprised 53 events. Thirty patients (17.0%) died: 21 (70.0%) from disease-related (lymphoma) causes, 4 (13.3%) from treatment-related causes, and 5 (16.7%) from other causes. Causes of death were classified by retrospective adjudication of the medical record by the treating team.

### Baseline PET/CT parameters

**Figure 2:**
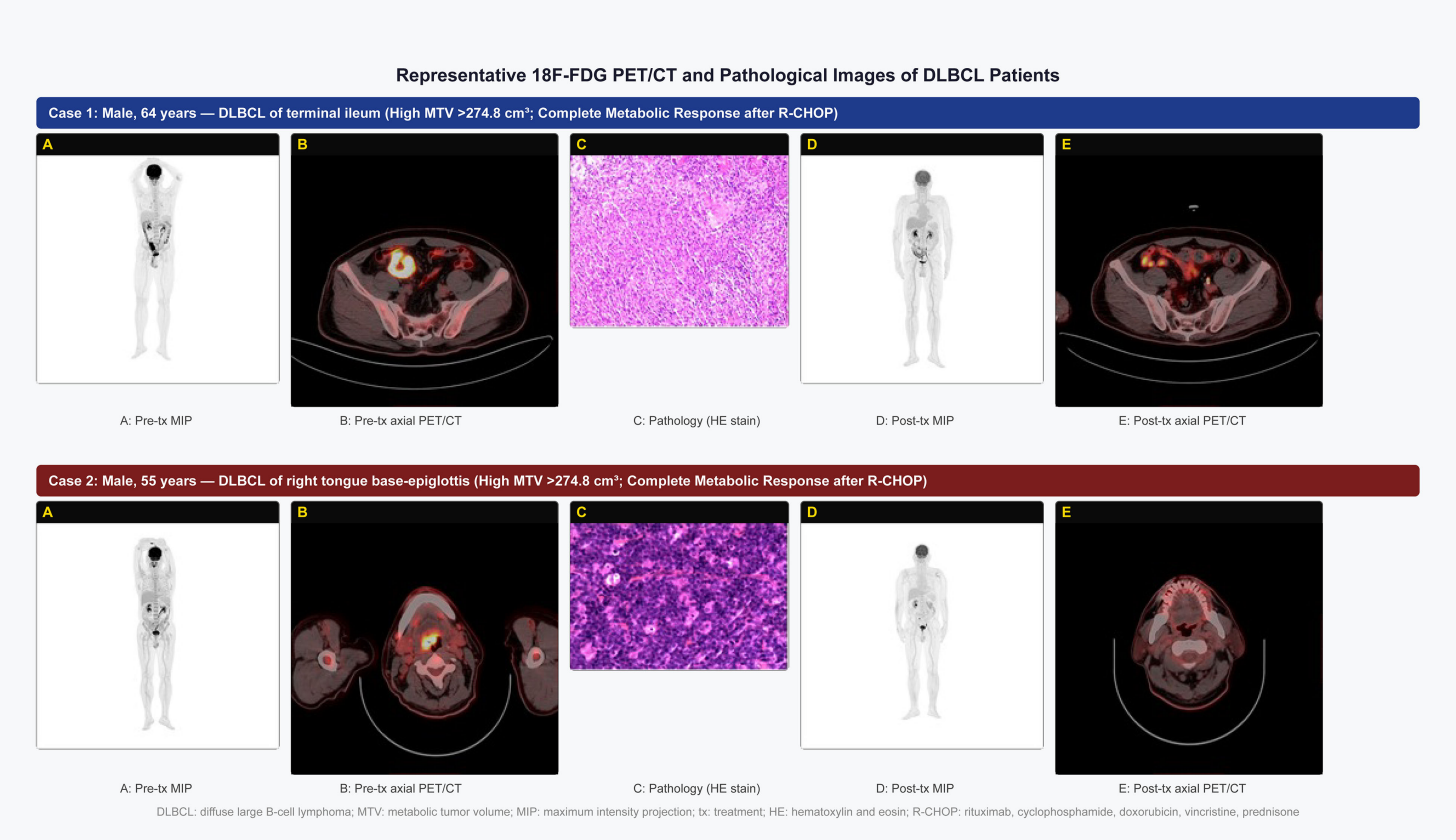
Representative ^18^F-FDG PET/CT and pathological images of two patients with diffuse large B-cell lymphoma (DLBCL) who achieved complete metabolic response after first-line R-CHOP immunochemotherapy (methodological illustration of the segmentation workflow; not presented as evidence of the prognostic association). Case 1 (upper panel): A 64-year-old man with primary DLBCL of the terminal ileum. Case 2 (lower panel): A 55-year-old man with primary DLBCL of the right tongue base–epiglottis. In both cases, baseline ^18^F-FDG PET/CT demonstrated a focal mass with markedly elevated FDG uptake (metabolic tumor volume [MTV] >274.8 cm³, the prognostically significant threshold identified in the present study). Post-treatment PET/CT confirmed complete metabolic response (Deauville score 1–2), with no residual abnormal FDG activity at the primary site. **(A)** Pre-treatment maximum-intensity-projection (MIP) image showing a solitary hypermetabolic lesion. **(B)** Pre-treatment axial PET/CT fusion image of the primary lesion with intense FDG uptake. **(C)** Hematoxylin and eosin (HE)-stained pathological section demonstrating diffuse proliferation of large atypical B-lymphocytes with prominent nucleoli, high nuclear-to-cytoplasmic ratio, and frequent mitotic figures, consistent with DLBCL. **(D)** Post-treatment MIP image showing resolution of the hypermetabolic lesion. **(E)** Post-treatment axial PET/CT fusion image at the primary site confirming absence of residual metabolic activity.

The median baseline SUVmax was 16.55 (IQR, 12.90-22.62), and the median SUVmean was 8.15 (IQR, 5.80-10.90). The median MTV was 186.40 cm³ (IQR, 95.17-408.60), and the median TLG was 1,524.80 g (IQR, 710.52-3,818.93). MTV and TLG were strongly correlated (Spearman r = 0.923; P < 0.001), whereas the correlation between MTV and SUVmax was negligible (r = 0.069; P = 0.366). Representative baseline and post-treatment PET/CT images alongside pathological sections from two patients with high-MTV DLBCL are presented in **Figure 2** as a methodological illustration of the volumetric segmentation workflow; these cases are intended to demonstrate the imaging methodology and are not presented as evidence of the prognostic association.

### ROC analysis and optimal cutoff determination

**Figure 3:**
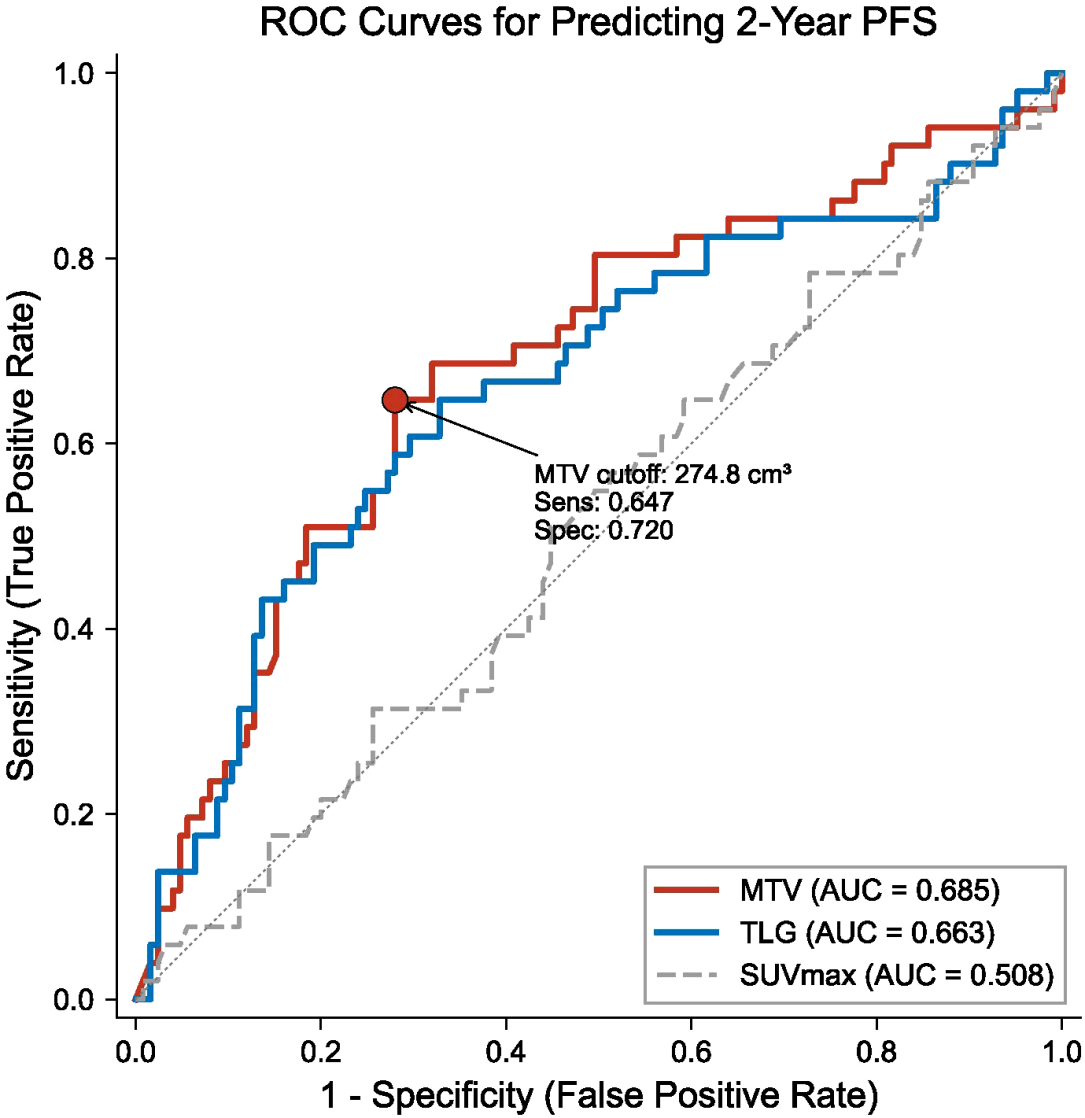
Receiver operating characteristic (ROC) curves for MTV, TLG, and SUVmax in predicting 2-year progression-free survival events. MTV demonstrated the highest discriminatory capacity (AUC = 0.685), followed by TLG (AUC = 0.663) and SUVmax (AUC = 0.508). The optimal MTV cutoff of 274.8 cm³ (sensitivity = 0.647, specificity = 0.720) is indicated by the red point.

ROC analysis demonstrated that MTV had the highest discriminatory capacity for predicting 2-year PFS events, with an AUC of 0.685 (95% CI, 0.598-0.772), followed by TLG (AUC = 0.663; 95% CI, 0.573-0.753) and SUVmax (AUC = 0.508; 95% CI, 0.410-0.606). The optimal cutoff value for MTV was 274.8 cm³ (sensitivity, 0.647; specificity, 0.720), and for TLG was 1,886.3 g (sensitivity, 0.647; specificity, 0.672) (**Figure 3**). Pairwise DeLong comparison confirmed that MTV and TLG each discriminated 2-year PFS events significantly better than SUVmax (P = 0.006 and P = 0.003, respectively), whereas MTV and TLG did not differ significantly (P = 0.25). Using the MTV cutoff, patients were stratified into a high-MTV group (n = 67, 38.1%) and a low-MTV group (n = 109, 61.9%).

### Survival outcomes by MTV and TLG groups

**Figure 4:**
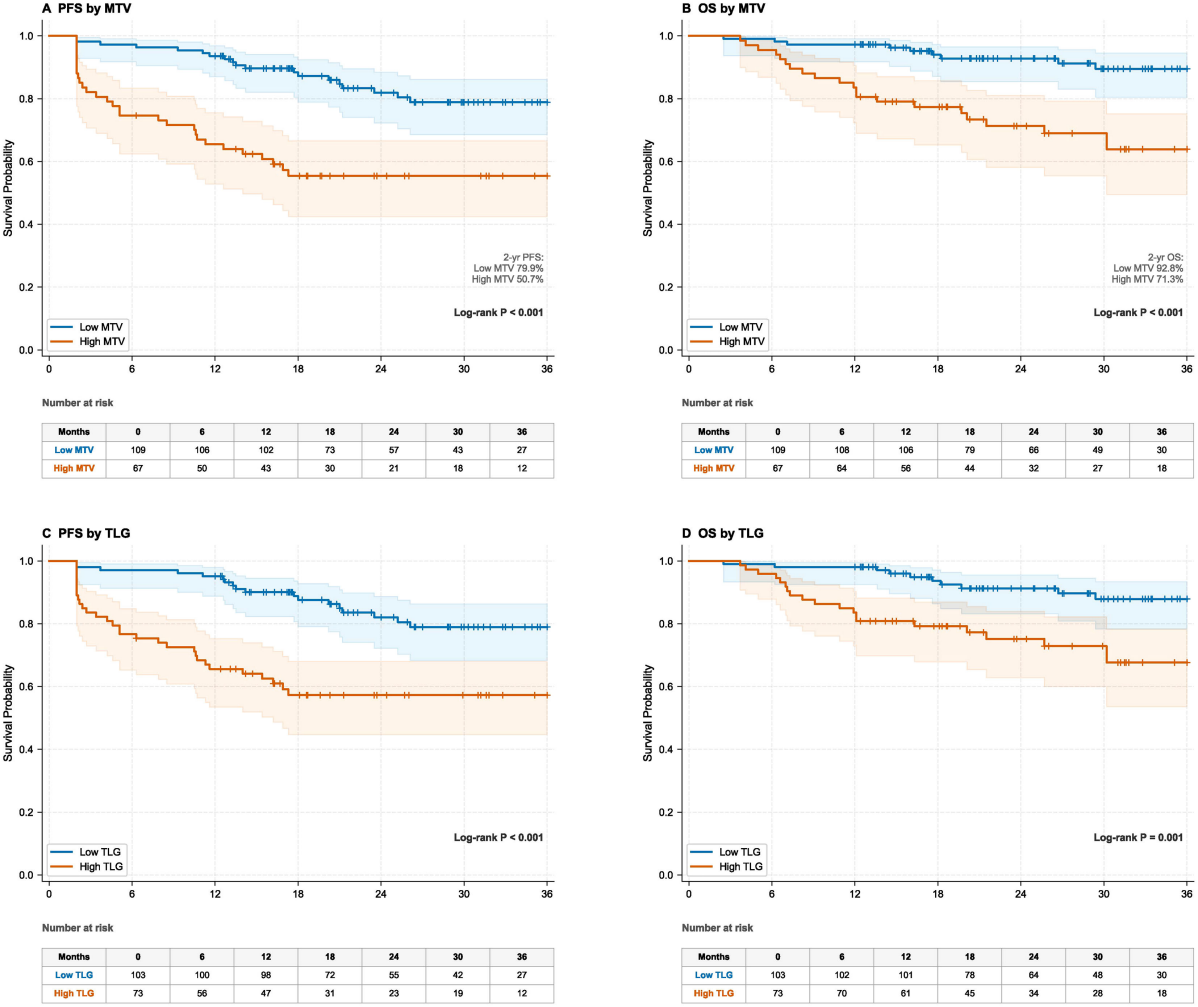
Kaplan-Meier survival curves stratified by MTV and TLG groups, with number-at-risk tables at 0, 6, 12, 18, 24, 30, and 36 months beneath each panel. **(A)** Progression-free survival by MTV group. **(B)** Overall survival by MTV group. **(C)** Progression-free survival by TLG group. **(D)** Overall survival by TLG group. P values are from the log-rank test.

Kaplan-Meier analysis revealed markedly divergent survival trajectories between MTV groups (**Figure 4**). Patients with high MTV (>274.8 cm³) had significantly inferior 2-year PFS compared with those with low MTV (50.7% vs 79.9%; log-rank P < 0.001). Similarly, 2-year OS was substantially worse in the high-MTV group (71.3% vs 92.8%; log-rank P < 0.001). An analogous pattern was observed when patients were stratified by TLG: high TLG (>1,886.3 g) was associated with inferior 2-year PFS (54.7% vs 78.9%; P < 0.001) and 2-year OS (75.1% vs 91.2%; P = 0.001).

### Univariate and multivariate Cox regression analyses

**Figure 5:**
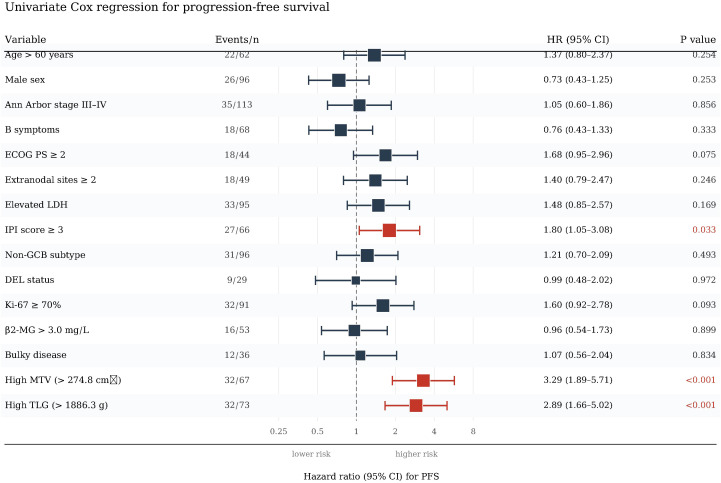
Forest plot of univariate Cox regression analysis for progression-free survival. Significant variables (P < 0.05) are shown in red. High MTV demonstrated the strongest association with inferior PFS (HR = 3.29; 95% CI, 1.89-5.71; P < 0.001).

Results of univariate Cox regression analyses for PFS and OS are presented in **Table 2** and **Figure 5**. In univariate analysis for PFS, high MTV (HR = 3.29; 95% CI, 1.89-5.71; P < 0.001), high TLG (HR = 2.89; 95% CI, 1.66-5.02; P < 0.001), IPI score ≥ 3 (HR = 1.80; 95% CI, 1.05-3.08; P = 0.033), ECOG PS ≥ 2 (HR = 1.68; 95% CI, 0.95-2.96; P = 0.075), and Ki-67 ≥ 70% (HR = 1.60; 95% CI, 0.92-2.78; P = 0.093) were identified as candidate prognostic factors (P < 0.10). Notably, SUVmax, cell-of-origin, DEL status, serum LDH, and β2-microglobulin did not achieve statistical significance for PFS in this cohort.

**Table 2:** Univariate and multivariate Cox regression analyses for progression-free survival and overall survival.

Variable	Univariate PFS HR (95% CI)	P	Multivariate PFS HR (95% CI)	P	Univariate OS HR (95% CI)	P	Multivariate OS HR (95% CI)	P
Age > 60 y	1.37 (0.80-2.37)	0.254	–	–	1.20 (0.58-2.50)	0.619	–	–
Male sex	0.73 (0.43-1.25)	0.253	–	–	0.58 (0.28-1.19)	0.136	–	–
Stage III-IV	1.05 (0.60-1.86)	0.856	–	–	1.10 (0.51-2.34)	0.813	–	–
B symptoms	0.76 (0.43-1.33)	0.333	–	–	0.62 (0.29-1.37)	0.239	–	–
ECOG PS ≥ 2	1.68 (0.95-2.96)	0.075	—	—	1.26 (0.58-2.76)	0.557	–	–
Extranodal ≥ 2	1.40 (0.79-2.47)	0.246	–	–	1.10 (0.50-2.39)	0.818	–	–
Elevated LDH	1.48 (0.85-2.57)	0.169	–	–	1.84 (0.86-3.93)	0.117	–	–
IPI ≥ 3	1.80 (1.05-3.08)	0.033	1.65 (0.96-2.82)	0.071	1.38 (0.67-2.83)	0.378	–	–
Non-GCB	1.21 (0.70-2.09)	0.493	–	–	0.83 (0.41-1.70)	0.618	–	–
DEL	0.99 (0.48-2.02)	0.972	–	–	0.78 (0.27-2.24)	0.648	–	–
Ki-67 ≥ 70%	1.60 (0.92-2.78)	0.093	—	—	1.51 (0.73-3.14)	0.269	–	–
β2-MG > 3.0	0.96 (0.54-1.73)	0.899	–	–	0.94 (0.43-2.06)	0.881	–	–
Bulky disease	1.07 (0.56-2.04)	0.834	–	–	1.16 (0.50-2.72)	0.724	–	–
High MTV	3.29 (1.89-5.71)	<0.001	3.16 (1.82-5.50)	<0.001	4.25 (1.95-9.29)	<0.001	4.25 (1.95-9.29)	<0.001
High TLG	2.89 (1.66-5.02)	<0.001	Excluded†	–	3.26 (1.52-6.96)	0.002	Excluded†	–

†Excluded from multivariate model due to collinearity with MTV (Spearman r = 0.923).

HR, hazard ratio; CI, confidence interval; PFS, progression-free survival; OS, overall survival. PFS is a composite endpoint (progression, relapse, or death from any cause; 53 events). Multivariable estimates reflect the final model after backward elimination (P > 0.10 for removal); covariates not retained are shown as —.

For OS, only high MTV (HR = 4.25; 95% CI, 1.95-9.29; P < 0.001) and high TLG (HR = 3.26; 95% CI, 1.52-6.96; P = 0.002) demonstrated significant prognostic associations in univariate analysis.

In multivariate Cox regression for PFS incorporating all variables with P < 0.10 from univariate analysis (excluding TLG to avoid collinearity with MTV), high MTV was the only statistically significant independent predictor of inferior PFS (adjusted HR = 3.16; 95% CI, 1.82-5.50; P < 0.001); IPI score ≥ 3 retained only a borderline, non-significant association after backward elimination (HR = 1.65; 95% CI, 0.96-2.82; P = 0.071), while ECOG PS ≥ 2 and Ki-67 ≥ 70% did not remain in the final model (**Table 2**). The bootstrap optimism-corrected hazard ratio for high MTV was 3.10 (95% CI, 1.95-6.05), indicating minimal overfitting. When the Youden-optimal cutoff was instead re-derived within each of the 1,000 bootstrap replications, the re-derived threshold spanned a comparatively wide range (median 267 cm³; IQR, 188-271), consistent with regarding the cutoff as optimism-prone and MTV as a continuous risk modifier rather than a fixed binary threshold. The optimism-corrected point estimate for the high-MTV hazard ratio was 2.93; the accompanying range, 2.65-3.53, is the percentile spread (dispersion) of the 1,000 bootstrap-corrected point estimates and is not a confidence interval. Because an optimism correction shifts the point estimate without reducing the sampling variability of the estimator, the corresponding 95% confidence interval for the corrected hazard ratio remains comparable in width to that of the apparent estimate (3.29; 95% CI, 1.89-5.71). The close agreement between the fixed-cutoff (3.10) and re-derived-cutoff (2.93) point estimates confirms that data-driven threshold selection did not materially inflate the estimate. When first-line regimen (R-CHOP vs intensified) and consolidation radiotherapy were added as covariates, high MTV remained robustly prognostic (adjusted HR = 3.18; 95% CI, 1.82-5.55; P < 0.001), and neither regimen (HR 1.12; P = 0.74) nor radiotherapy (HR 0.77; P = 0.43) was independently associated with PFS. In the R-CHOP-only subgroup (n = 136), high MTV retained independent prognostic significance (adjusted HR = 2.42; 95% CI, 1.31-4.47; P = 0.005). The association was also robust to imaging platform: the adjusted hazard ratio for high MTV was essentially unchanged when scanner model was included as a covariate (HR = 3.30; 95% CI, 1.90-5.74) or used as a stratification factor (HR = 3.28; 95% CI, 1.88-5.71), with no significant MTV-by-scanner interaction (P = 0.30; **Supplementary Table 2**). Modelled continuously, each 1-SD increase in log-transformed MTV was associated with higher progression risk (HR = 1.63; 95% CI, 1.27-2.11; P < 0.001), with evidence of a non-linear dose-response relationship on restricted cubic spline analysis (P for non-linearity = 0.002). For OS, given only 30 events, the analysis was restricted as prespecified to a single predictor; high MTV was associated with inferior OS (HR = 4.25; 95% CI, 1.95-9.29; P < 0.001). This OS analysis is exploratory and hypothesis-generating, the wide confidence interval reflecting limited statistical power; MTV should not be regarded as a definitively established independent predictor of OS on the basis of these data.

### Incremental prognostic value of MTV over the IPI

**Figure 6:**
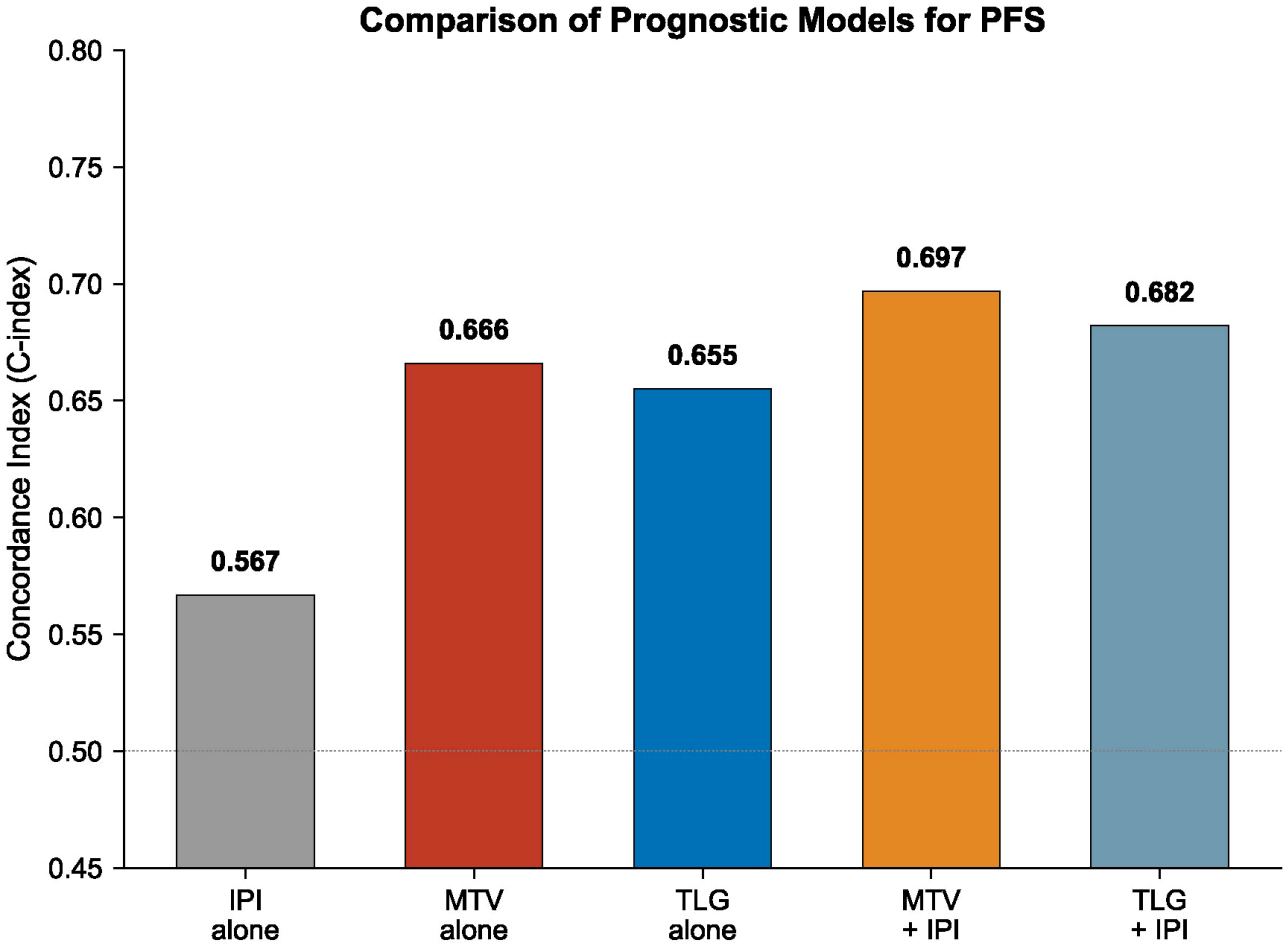
Comparison of concordance indices (C-indices) across prognostic models for progression-free survival prediction. The combined MTV + IPI model yielded the highest C-index (0.697), outperforming IPI alone (0.567) and MTV alone (0.666).

To evaluate whether MTV adds prognostic information beyond the conventional IPI, we compared the discriminatory performance of several models using the C-index (**Figure 6**; **Table 3**). The IPI alone yielded a C-index of 0.567 (95% CI, 0.491–0.643) for PFS prediction, whereas MTV alone achieved 0.666 (95% CI, 0.592–0.740). TLG alone yielded a C-index of 0.655 (95% CI, 0.576–0.728). The combined MTV-IPI model demonstrated a C-index of 0.697 (95% CI, 0.625–0.769), representing a numerical improvement over both individual components that warrants confirmation in independent cohorts. TLG combined with IPI yielded a C-index of 0.682 (95% CI, 0.608–0.756). A full multivariate model incorporating MTV, ECOG PS, IPI, and Ki-67 achieved the highest C-index of 0.710 (95% CI, 0.639–0.781), although the incremental gain over the parsimonious MTV-IPI model was modest. These findings indicate that MTV provides substantial incremental prognostic value when integrated with established clinical risk factors. After bootstrap internal validation optimism was minimal, with optimism-corrected C-indices of 0.564 (IPI), 0.666 (MTV), and 0.692 (MTV+IPI). The improvement in discrimination from adding MTV to the IPI was statistically significant (bootstrap difference in C-index 0.129; 95% CI, 0.053-0.199; P < 0.001), whereas the gain of the full model over the parsimonious MTV+IPI model was not (difference 0.020; 95% CI, -0.003 to 0.053; P = 0.10). Adding MTV to the IPI also yielded a continuous NRI of 0.67 (95% CI, 0.24-0.95) and an IDI of 0.075 (95% CI, 0.013-0.175; P < 0.001), confirming improved risk reclassification. For reference, an IMPI-style model (continuous MTV, age, and stage) fitted to our cohort achieved a C-index of 0.667, comparable to but not exceeding the MTV+IPI model.

**Table 3:** Comparison of prognostic model performance.

Model	C-index (95% CI) for PFS	Variables included
IPI alone	0.567 (0.491-0.643)	IPI score ≥ 3
MTV alone	0.666 (0.592-0.740)	MTV > 274.8 cm³
TLG alone	0.655 (0.576-0.728)	TLG > 1886.3 g
MTV + IPI	0.697 (0.625-0.769)	MTV > 274.8 cm³, IPI score ≥ 3
TLG + IPI	0.682 (0.608-0.756)	TLG > 1886.3 g, IPI score ≥ 3
Full multivariate model	0.710 (0.639-0.781)	MTV, ECOG, IPI, Ki-67

C-index, concordance index (with 95% bootstrap confidence intervals); PFS, progression-free survival; IPI, International Prognostic Index; MTV, metabolic tumor volume; TLG, total lesion glycolysis; ECOG, Eastern Cooperative Oncology Group. Optimism-corrected C-indices (1,000 bootstrap replications) were 0.564 (IPI), 0.666 (MTV), 0.654 (TLG), 0.692 (MTV+IPI), 0.675 (TLG+IPI), and 0.691 (full model). The increase in C-index from adding MTV to the IPI was significant (difference 0.129; 95% CI, 0.053-0.199; P < 0.001), whereas the full model did not significantly exceed the MTV+IPI model (difference 0.020; 95% CI, -0.003 to 0.053; P = 0.10). Adding MTV to the IPI yielded a continuous net reclassification improvement of 0.67 (95% CI, 0.24-0.95) and an integrated discrimination improvement of 0.075 (95% CI, 0.013-0.175; P < 0.001).

### Subgroup analyses

**Figure 7:**
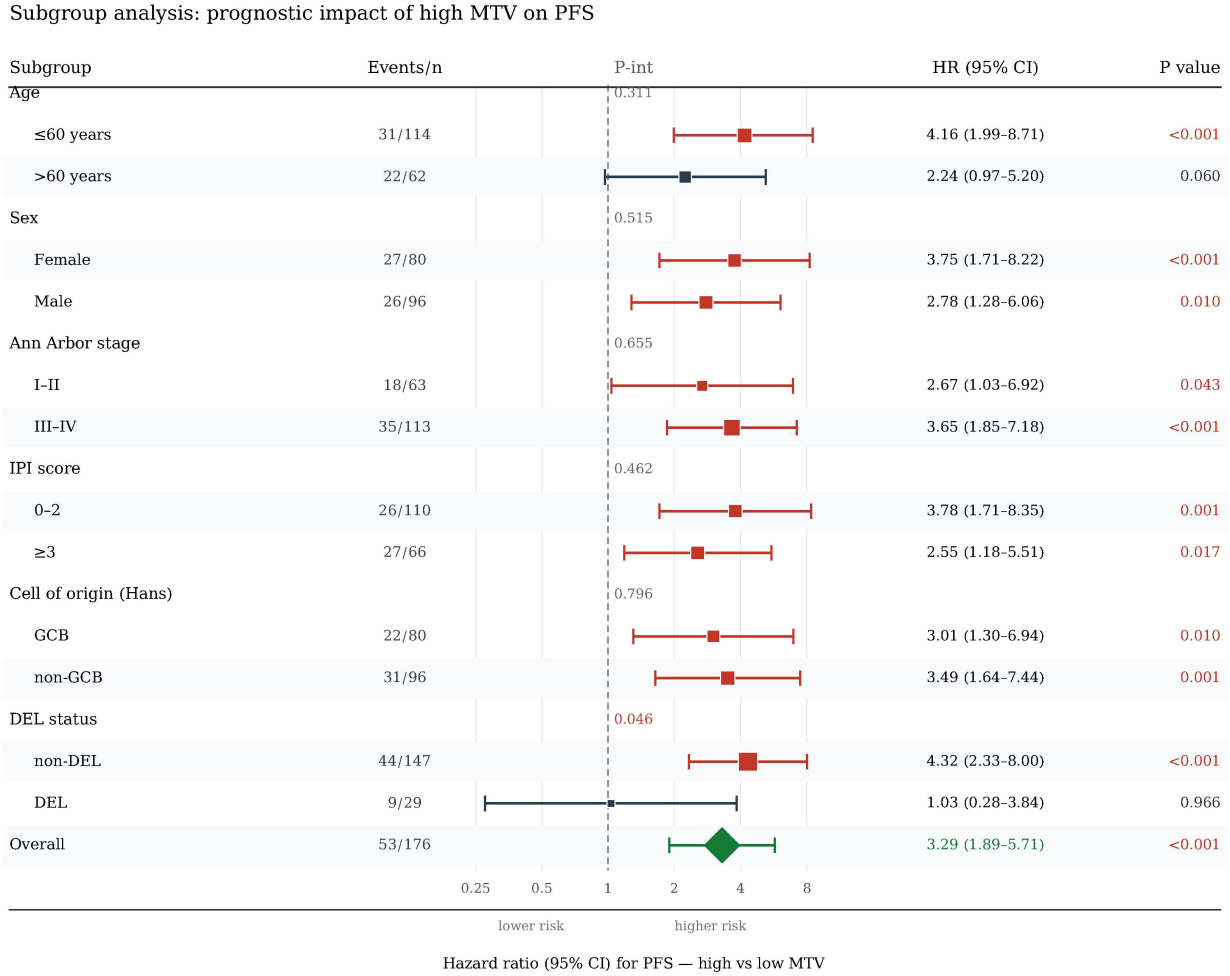
Subgroup analysis of the prognostic impact of high MTV on progression-free survival across predefined clinical strata. The diamond marker represents the overall cohort. A significant interaction was observed with double-expressor lymphoma (DEL) status (P for interaction = 0.046): the prognostic effect of high MTV was significant in non-DEL patients but not in the small DEL subgroup.

To assess the robustness and generalizability of MTV-based risk stratification, we performed prespecified exploratory subgroup analyses across clinically relevant strata (**Figure 7**). High MTV consistently predicted inferior PFS in both the GCB subgroup (2-year PFS: 53.2% vs 78.9%; P = 0.007; n = 80) and the non-GCB subgroup (49.3% vs 80.8%; P = 0.001; n = 96). The prognostic impact of MTV remained significant across IPI risk categories: in patients with IPI 0-2, high MTV was associated with a 2-year PFS of 56.1% versus 83.3% for low MTV (P < 0.001; n = 110), and in patients with IPI 3-5, the corresponding rates were 43.8% versus 73.7% (P = 0.014; n = 66). Similarly, MTV retained its discriminatory capacity in both early-stage (stage I-II; P = 0.035; n = 63) and advanced-stage disease (stage III-IV; P < 0.001; n = 113). Across most strata MTV retained prognostic significance; however, a significant interaction was observed with DEL status (P for interaction = 0.046): the prognostic effect of high MTV was strong in non-DEL patients (HR = 4.32; 95% CI, 2.33-8.00; P < 0.001; n = 147) but was not evident in the small DEL subgroup (HR = 1.03; 95% CI, 0.28-3.84; P = 0.966; n = 29). This subgroup observation is hypothesis-generating and is interpreted with caution (see Discussion).

## Discussion

In this retrospective analysis of 176 patients with newly diagnosed DLBCL treated with R-CHOP-based immunochemotherapy, we demonstrate that baseline MTV, measured with a standardized 41% SUVmax threshold on ^18^F-FDG PET/CT, is a robust independent predictor of both PFS and OS. Patients with high MTV (>274.8 cm³) experienced approximately three-fold higher risk of disease progression (HR = 3.16) and more than four-fold higher risk of death (HR = 4.25) compared with those with low MTV. Importantly, MTV retained its prognostic significance after adjustment for conventional clinical variables and across all examined subgroups, including cell-of-origin subtypes and IPI risk strata. The integration of MTV with IPI improved the C-index from 0.567 to 0.697, underscoring the complementary nature of volumetric metabolic assessment and traditional clinical prognostication.

These findings align with and extend a growing body of evidence supporting the prognostic utility of PET-derived volumetric parameters in DLBCL. The study by Zhang et al. (12), analyzing 262 DLBCL patients, demonstrated that baseline MTV is the optimal PET prognostic parameter with an AUC of 0.652 for predicting outcomes and that MTV significantly improves IPI stratification regardless of risk category, findings that closely parallel our observations. Alderuccio et al. (13) further demonstrated in the LOTIS-2 clinical trial that MTV with a cutoff of 96 mL exhibited the highest predictive performance for treatment failure, PFS (HR = 2.68), and OS (HR = 3.09), reinforcing MTV as a robust risk stratification tool across diverse clinical settings. The recently published IMPI (15), derived from the PETRA database, combines MTV with age and stage to yield a composite score with a C-index of 0.674 to 0.696 for PFS and OS. Our finding that the combined MTV-IPI model achieves a C-index of 0.697 is comparable to the IMPI performance and supports the notion that integrating volumetric and clinical data yields superior risk stratification.

A notable observation in our study is the relatively modest AUC of MTV for predicting 2-year PFS events (0.685), which, while superior to both TLG (0.663) and SUVmax (0.508), falls below values reported in certain validation cohorts (21). This discrepancy may partly reflect the inherent limitation of ROC analysis applied to time-to-event data, where it does not account for censoring; in contrast, the robust prognostic associations observed in Cox regression and Kaplan-Meier analyses provide a more appropriate assessment of MTV discriminatory capacity (22). Furthermore, the substantially larger AUC difference between MTV and SUVmax is consistent with the comprehensive prognostic review by Duminuco et al. (23), who confirmed that the majority of studies report no significant predictive ability for baseline SUVmax, whereas volumetric parameters such as MTV demonstrate consistent and reproducible prognostic significance. This divergence underscores a conceptual distinction. SUVmax captures peak metabolic intensity at a single voxel, whereas MTV reflects the aggregate three-dimensional disease burden. The latter appears to encode more clinically relevant biological information, consistent with the negligible correlation between MTV and SUVmax observed in our cohort, which indicates that MTV captures a distinct biological dimension of tumor burden beyond metabolic intensity alone.

The optimal MTV cutoff of 274.8 cm³ identified in our analysis falls within the broad range reported in the literature,23 though the substantial variability in optimal cutoffs across studies—from 96 cm³ in the LOTIS-2 trial13 to over 400 cm³ in other series—remains a principal barrier to clinical translation, reflecting differences in patient populations, segmentation methodology, and statistical approaches to threshold selection (17, 19). The 2024 JNM international benchmark dataset (19) represents a critical step toward standardizing TMTV measurement and establishing reproducible reference values. A further methodological consideration concerns PET/CT system heterogeneity: our cohort was imaged on two PET/CT systems with differing detector configurations and sensitivities. Although a uniform 41% SUVmax threshold was applied to limit inter-system variability, differences in spatial resolution, noise, and partial-volume effects can systematically influence volumetric quantification, and formal cross-vendor EARL harmonization was not performed. Multi-vendor acquisition may broaden generalizability relative to single-scanner studies but also introduces measurement heterogeneity; consequently, the absolute 274.8 cm³ cutoff identified here may not transfer directly to cohorts scanned on other systems. A scanner-adjusted and scanner-stratified sensitivity analysis was performed to evaluate the robustness of the high-MTV association with PFS across the two systems, with no significant effect heterogeneity (**Supplementary Tables 1**, **S2**). These considerations reinforce the value of the international TMTV benchmark initiative (19) and of EARL-harmonized acquisition for reproducible, transferable thresholds. Our use of the 41% SUVmax threshold aligns with the EANM-recommended methodology and the approach adopted in the majority of recent large-scale studies, facilitating comparability. Critically, because the 274.8 cm³ threshold was derived by maximizing the Youden index within the same cohort used for survival analysis, it should be regarded as an optimism-prone, upper-bound estimate that requires external validation before any clinical application.

TLG, which incorporates both volumetric and metabolic intensity data, demonstrated comparable but slightly inferior prognostic performance to MTV in our cohort. This finding is consistent with the observation by Liao et al. (24) that TLG may serve as an independent predictor in some settings, and with the strong correlation between MTV and TLG (r = 0.923) that limits the independent contribution of TLG in multivariate models. Practically, this correlation suggests that the additional computational step of calculating TLG may offer limited incremental value over MTV alone, a finding with implications for streamlining clinical workflow. Notably, at the 274.8 cm³ threshold the moderate sensitivity (0.647) and specificity (0.720) correspond to an approximately 35% false-negative rate for 2-year PFS events, underscoring that MTV is best applied as a continuous, complementary risk modifier rather than a strict binary classifier at the individual-patient level. The slightly lower stratification performance of TLG relative to MTV probably reflects that multiplying volume by SUVmean introduces additional measurement variability while reducing the parameter to a composite dominated by volume; once tumor burden is accounted for, the SUVmean component adds little independent prognostic information, supporting MTV as the more robust and reproducible volumetric biomarker.

The subgroup analyses provide particularly informative insights. The consistent prognostic impact of MTV across GCB and non-GCB subtypes is noteworthy, as cell-of-origin and emerging genetic classifications24 have been variably associated with outcome in the rituximab era (8, 25). Our data suggest that MTV captures a dimension of disease biology that transcends molecular subtype classification, possibly reflecting the total proliferating and metabolically active tumor mass regardless of the underlying pathobiology. Similarly, the sustained discriminatory capacity of MTV within both low-risk (IPI 0-2) and high-risk (IPI 3-5) strata echoes the finding by Alderuccio et al. (13) and Ikeda et al. (21) that MTV provides additive prognostic information beyond the IPI, and further supports the integration of MTV into composite risk models such as the IMPI (15). The significant MTV-by-DEL interaction nonetheless merits comment. The apparent loss of MTV prognostic value among DEL patients may reflect a genuine biological effect, whereby MYC/BCL2 co-expression becomes a dominant driver of adverse outcome that overrides tumor-burden effects, or, more likely given the small DEL subgroup (n = 29), limited statistical power. This observation is hypothesis-generating and warrants confirmation in larger cohorts enriched for DEL.

Several limitations of this study merit acknowledgment. First, the retrospective single-center design introduces potential selection and information biases that limit the generalizability of our findings. Second, the median follow-up of 26.4 months, while adequate for assessing 2-year survival endpoints, precludes evaluation of longer-term outcomes, and the number of events, particularly for OS, limits the statistical power of multivariate analyses. Third, the absence of an independent external validation cohort prevents confirmation of the identified MTV cutoff and the performance of the combined MTV-IPI model. Fourth, although mitigated by the use of a standardized threshold and consensus dual-reader assessment, inter-reader variability remains an inherent limitation of volumetric PET analysis; a quantitative inter-reader agreement metric could not be computed from the retained consensus dataset. Fifth, the rapidly evolving molecular classification of DLBCL27 may influence the interpretation of volumetric PET parameters, and future studies should explore the interaction between genetic subtypes and MTV-based risk stratification. Relatedly, patients were classified using the WHO 2016 framework in routine use during enrolment; reclassification under the WHO 5th edition (2022) (26) or the International Consensus Classification (2022) (27), which refine large B-cell lymphoma entities (for example, high-grade B-cell lymphoma with 11q aberrations, EBV-positive DLBCL, and large B-cell lymphoma with IRF4 rearrangement), was not undertaken and may affect cohort composition. Sixth, interim PET response metrics were not integrated into the present prognostic models; nonetheless, baseline MTV (a measure of initial tumor burden) and interim PET response (Deauville score, ΔSUVmax) are likely complementary rather than redundant, capturing pre-treatment disease extent and early chemosensitivity, respectively, and their combination has been shown to improve risk prediction (28). We also did not apply more advanced radiomics or machine-learning approaches that may capture additional spatial and textural tumor information (29).

Looking ahead, several directions warrant further exploration. Prospective multicenter studies with standardized acquisition and segmentation protocols, ideally building on the 2024 JNM benchmark framework (19), are essential to validate MTV cutoffs and confirm the incremental value of MTV-augmented prognostic models. The integration of MTV with molecular subtyping, including the recently highlighted DEL phenotype (9, 30), interim PET response metrics such as the Deauville score and ΔSUVmax,28 and emerging radiomics signatures may enable truly personalized risk stratification. In particular, circulating tumor DNA, which enables non-invasive genotyping and minimal-residual-disease monitoring and has been shown to complement metabolic imaging including interim ΔSUVmax (31), represents a promising biomarker to integrate with baseline MTV for dynamic, personalized risk assessment. Finally, linking MTV-based risk assessment to risk-adapted therapeutic strategies, including the potential role of intensified frontline regimens for patients with high MTV, represents a critical translational priority.

## Conclusions

In this retrospective cohort of 176 DLBCL patients treated with R-CHOP-based immunochemotherapy, baseline MTV derived from ^18^F-FDG PET/CT using a standardized 41% SUVmax threshold was a robust, independent prognostic biomarker for PFS in this single-center cohort that substantially enhanced risk discrimination beyond the conventional IPI, while its association with OS remained exploratory. The combined MTV-IPI model demonstrated a C-index of 0.697 (95% CI, 0.625–0.769), representing a meaningful improvement over IPI alone. These findings were consistent across cell-of-origin subtypes, disease stages, and IPI risk categories. Because the cutoff was internally derived and optimism-prone, external validation is required before clinical use; prospective multicenter validation with standardized measurement protocols and the exploration of MTV-guided risk-adapted treatment strategies represent essential next steps toward clinical implementation.

## Data Availability

The original contributions presented in the study are included in the article/**Supplementary Material**. Further inquiries can be directed to the corresponding author.
