# Supplementary material for: Metabolic tumor volume and total lesion glycolysis for predicting prognosis in diffuse large B-cell lymphoma: a retrospective PET/CT study
Source: Front Oncol. 2026 Jul 31;16:1862170. doi: 10.3389/fonc.2026.1862170 (PMC13472934; doi:10.3389/fonc.2026.1862170)
Supplement: Supplementary file 1 [file DataSheet1.docx]

**Supplementary Materials**

Metabolic Tumor Volume and Total Lesion Glycolysis for Predicting Prognosis in Diffuse Large B-Cell Lymphoma: A Retrospective PET/CT Study

**Supplementary Table S1.** **PET/CT acquisition and image-reconstruction parameters by PET/CT system**

| **Parameter** | **United Imaging Healthcare uEXPLORER (n = 92)** | **GE Discovery VCT (n = 84)** |
| --- | --- | --- |
| Reconstruction algorithm | OSEM (PSF + TOF) | OSEM, VPFX (PSF + TOF) |
| Reconstruction matrix | 192 × 192 | 256 × 256 |
| Voxel size (mm) | 3.13 × 3.13 × 2.68 | 2.73 × 2.73 × 2.79 |
| Iterations × subsets | 2 × 20 | 3 × 16 |
| Post-reconstruction filter | Gaussian, 3 mm FWHM | Gaussian, 4 mm FWHM |
| Point-spread-function (PSF) | Yes | Yes |
| Time-of-flight (TOF) | Yes | Yes |
| Attenuation correction | CT-based | CT-based |
| Segmentation threshold | 41% SUVmax | 41% SUVmax |

The values shown are the institutional clinical reconstruction protocol applied on each platform, as confirmed by the authors against the imaging records, and are consistent with the corresponding vendor-recommended settings and EANM procedure guidelines (version 2.0). Scanner-specific reconstruction settings are shown for the two PET/CT systems. Point-spread-function and time-of-flight modelling were applied where available according to the vendor-specific clinical protocol, and a single uniform 41% SUVmax threshold was used for volumetric segmentation across both systems to mitigate inter-system variability. Formal cross-vendor EARL harmonization was not performed, and a single uniform 41% SUVmax threshold was used for volumetric segmentation across all scanners to mitigate inter-system variability. Formal cross-vendor EARL harmonization was not performed (a limitation discussed in the main text). OSEM, ordered-subset expectation maximization; FWHM, full width at half maximum; SUVmax, maximum standardized uptake value; EANM, European Association of Nuclear Medicine.

**Supplementary Table S2. Sensitivity of the high-MTV–PFS association to PET/CT system**

(a) High MTV (>274.8 cm³) versus low MTV, adjusted for or stratified by scanner platform (Cox proportional-hazards models; outcome, progression-free survival).

| **Model** | **Hazard ratio (95% CI)** | **P value** |
| --- | --- | --- |
| High MTV, unadjusted | 3.29 (1.89–5.71) | <0.001 |
| High MTV + scanner model (covariate) | 3.30 (1.90–5.74) | <0.001 |
| High MTV, stratified by scanner model | 3.28 (1.88–5.71) | <0.001 |

(b) Descriptive within-platform high-MTV hazard ratios (exploratory; limited events per scanner).

| **Scanner platform** | **Patients (n)** | **PFS events (n)** | **High MTV (n)** | **Hazard ratio (95% CI)** | **P value** |
| --- | --- | --- | --- | --- | --- |
| United Imaging Healthcare uEXPLORER | 92 | 24 | 35 | 6.51 (2.05–20.71) | 0.001 |
| GE Discovery VCT | 84 | 29 | 32 | 1.74 (0.58–5.19) | 0.320 |

The global test for an MTV-by-scanner interaction was not significant (likelihood-ratio test P = 0.30), indicating no statistically detectable heterogeneity of the MTV effect across platforms. Within-platform estimates are directionally concordant (all hazard ratios >1) but imprecise owing to the small number of events per scanner and are presented for descriptive purposes only. CI, confidence interval; MTV, metabolic tumor volume; PFS, progression-free survival.

**Supplementary Note. Bootstrap optimism correction with re-derivation of the MTV cutoff**

In the primary analysis reported in the main text, the optimism-corrected hazard ratio for high MTV (3.10; 95% CI, 1.95–6.05) was obtained by 1,000 bootstrap replications with the Youden-optimal MTV cutoff held fixed at the value derived in the full cohort (274.8 cm³). To additionally account for the optimism introduced by data-driven threshold selection, the Youden-optimal cutoff was re-derived de novo within each of 1,000 bootstrap replications. Across replications the re-derived cutoff spanned a comparatively wide range (median 267 cm³; interquartile range, 188–271 cm³), and the resulting optimism-corrected point estimate for the high-MTV hazard ratio was 2.93. The accompanying range, 2.65–3.53, is the percentile spread (dispersion) of the 1,000 bootstrap-corrected point estimates and is not a confidence interval: an optimism correction shifts the point estimate but does not reduce the sampling variability of the estimator, so the corresponding 95% confidence interval for the corrected hazard ratio remains comparable in width to that of the apparent estimate (3.29; 95% CI, 1.89–5.71). The close agreement between the fixed-cutoff (3.10) and re-derived-cutoff (2.93) point estimates indicates that data-driven threshold selection did not materially inflate the apparent prognostic effect of MTV. Analyses were performed in R version 4.5.0 (packages survival, rms, and pROC).
